# Evaluation of Clinical, Biochemical, and Demographic Characteristics of Paediatric COVID-19 Patients Admitted to Dicle University Hospital

**DOI:** 10.7759/cureus.34171

**Published:** 2023-01-24

**Authors:** Cihan Akay, Özhan Orhan, Velat Şen

**Affiliations:** 1 Pediatrics, Erzurum Hınıs State Hospital, Erzurum, TUR; 2 Pediatrics, Mardin Artuklu University, Mardin, TUR; 3 Pulmonology, Dicle University School of Medicine, Diyarbakir, TUR

**Keywords:** fibrinojen, ferritin, clinical course, mortality, covid-19

## Abstract

Introduction and aim: In this study, we aim to determine how laboratory parameters were related to the clinical courses of patients admitted to the Dicle University Faculty of Medicine Department of Paediatrics and Paediatric Intensive Care Unit with COVID-19 diagnoses from March 2020 to November 2021.

Materials and method: Clinical, biochemical and demographic characteristics of 220 patients between 0 and 16 years old with COVID-19 diagnoses at admission were analysed retrospectively.

Results: We found that 57.3% of patients were male and 42.7% female, with a mean age of 107.8 ± 65.5 (range 1-192) months. Of the cases, 48.6% (n = 107) were asymptomatic, 35.5% (n = 78) were mild, 11.8% (n = 26) were moderately severe and 3.6% (n = 8) were severe. The patients’ site of admission, mortality rates, C reactive protein (CRP), lactate dehydrogenase (LDH), ferritin, and fibrinogen levels differed significantly (p < 0.001).

Conclusion: It is important to learn about the clinical course of the disease by accurately interpreting the results of blood parameters and appropriate imaging studies.

## Introduction

Coronaviruses are enveloped, positive-polarity single-stranded RNA viruses as a broad virus family causing disease presentations ranging from mild infections to more severe forms of infection, such as Middle East respiratory syndrome and severe acute respiratory syndrome (SARS) [1]. In December 2019, SARS-CoV-2 was identified as the most recent pathogen in a pneumonia epidemic, with Wuhan City, China, being the epicentre. The disease was denominated as coronavirus disease 2019 (COVID-19).

COVID-19 is a severe worldwide public health problem. SARS-CoV-2 infection can cause asymptomatic disease, mild upper respiratory tract infection, respiratory insufficiency or severe viral pneumonia, which can result in death [2,3]. The clinical and laboratory presentation of the paediatric age group reportedly differed from that of adults and had a milder course [4].

Although “real-time” reverse transcriptase polymerase chain reaction (RT-PCR) constitutes the main method to diagnose COVID-19, the test has a low sensitivity and false negative results are common. Because of this, the detection of the disease is usually late. Therefore, patients should be evaluated with clinical and biochemical findings and the results of thoracic computerised tomography [5].

In this paper, we aimed to foresee the course of the disease and take necessary measures by analysing the laboratory data of paediatric COVID-19 patients hospitalised at the Dicle University Faculty of Medicine Department of Paediatrics upon their positive PCR test results.

## Materials and methods

This retrospective study included 220 patients aged 0-16 with a positive COVID-19 PCR test result who received inpatient treatment at the Dicle University Faculty of Medicine Paediatric COVID-19 Clinic between 1 March 2020 and 22 December 2021. Dicle University Noninvasive Clinical Research Ethics Committee approval was obtained before commencing the study.

Inclusion criteria were having a COVID-19 diagnosis confirmed by a positive PCR test result, being 0-16 years old, and being treated and monitored as an inpatient.

Exclusion criteria were having a COVID-19 diagnosis unconfirmed by a PCR test, being older than 16 years, or having clinical information and biochemical results missing in the hospital information management system.

Demographic (sex, age), clinical (disease severity, admission complaint, admission unit and length of hospitalisation) and laboratory parameters of patients diagnosed and admitted to the hospital during the pandemic were examined retrospectively. Patient records were used to access the clinical and biochemical results. To this end, the patients’ blood count, biochemistry and coagulation parameters were compared.

Combined oropharyngeal/nasopharyngeal swab samples were obtained from all participants for COVID-19. They were studied at the laboratory using the Light Cyler 96 (ROCHE®, Basel, Switzerland) device and the DIAGNOVITAL® DIAGNO5plex NS SARS-CoV-2 Real Time PCR (İstanbul, Turkey) detection kits in line with the instructions of the manufacturer, resulting in positive or negative labelling according to manufacturers’ instructions.

Statistical analysis

Statistical Package for Social Sciences (SPSS) version 24 (IBM Corp., Armonk, NY, USA) software was used to conduct all statistical analyses. Percentage values were calculated for discrete variables. Two groups were compared with Student’s t-test or Mann-Whitney U test in the case of numerical variables, while categorical variables were compared with the help of the chi-square test. Student’s t-test was used to check the significance levels of correlation coefficients, and the two-sided level of significance was accepted as α < 0.05.

## Results

The analysis of the patients regarding sex distribution showed that 57.3% were male and 42.7% female, with a mean age of 107.8 ± 65.5 (range 1-192) months. The mean length of hospitalisation was 3.7 ± 3.1 (range 1-20) days.

The analysis of admission complaints revealed that 23.9% of patients had a fever, 22.5% a cough, 16.5% malaise, 5.5% vomiting, 12.4% a headache, 7.3% diarrhoea, 5% tachypnoea and 6% abdominal pain.

Five (2.3%) patients died at the end of the treatment period. Two hundred and three (92.7%) patients were hospitalised at the department of paediatrics and 16 (7.3%) in the COVID-19 paediatric intensive care unit.

The classification of the patients by disease severity demonstrated that 48.6% (n = 107) had an asymptomatic disease, 35.5% (n = 78) mild disease, 11.8% (n = 26) moderately severe disease and 3.6% (n = 8) severe disease. One patient who presented to the paediatric emergency department died.

The analysis of the laboratory results by the status of hospital discharge showed that the discharged patients had a mean C reactive protein (CRP) level of 1.28 ± 3.73, and the deceased patients had a mean CRP level of 8.78 ± 8.14. The discharged patients had a mean lactate dehydrogenase (LDH) level of 276.82 ± 120.32, and the deceased patients had a mean LDH level of 654.00 ± 279.22. The mean ferritin level of the discharged patients was 146.80 ± 448.47, and that of the deceased patients was 1269.03 ± 412.55. The mean fibrinogen levels of the discharged and deceased patients were 250.81 ± 113.05 and 427.33 ± 259.18, respectively. A significant difference was observed between the CRP, LDH, ferritin and fibrinogen levels of the discharged and deceased patients (p < 0.001) (Table 1).

**Table 1:** Relationship between patients' mortality status and laboratory results Data are expressed as mean standard deviation (SD) or frequencies (P) as appropriate. N; number of people,  A; average, WBC; white blood cell, ANC; absolute neutrophil count, ALC; absolute lymphocyte count, CRP; C reactive protein, LDH; lactate dehydrogenase, ALT; alanine transaminase, AST; aspartate transaminase, CK; creatine kinase

		N	A	SD	p
WBC	Discharged	195	7,23	3,58	0,694
	Exitus	5	7,89	6,62	
ANC	Discharged	195	3,57	2,73	0,159
	Exitus	5	5,38	5,42	
ALC	Discharged	195	2,81	2,24	0,198
	Exitus	5	1,51	1,50	
CRP	Discharged	193	1,28	3,73	0,001
	Exitus	5	8,78	8,14	
LDH	Discharged	192	276,82	120,32	0,001
	Exitus	5	654,00	279,22	
ALT	Discharged	206	34,08	20,24	0,008
	Exitus	5	58,76	23,23	
AST	Discharged	207	22,08	53,40	0,597
	Exitus	5	34,78	17,39	
Ferritin	Discharged	182	146,80	448,47	0,001
	Exitus	3	1269,03	412,55	
Fibronogen	Discharged	192	250,81	113,05	0,003
	Exitus	4	427,33	259,18	
D-Dimer	Discharged	185	1,75	6,31	0,901
	Exitus	4	1,36	0,72	
Hemoglobin	Discharged	193	12,55	1,69	0,369
	Exitus	5	11,86	1,19	
Platelet	Discharged	193	276,11	113,79	0,663
	Exitus	5	253,80	45,48	
Vitamin D	Discharged	78	18,22	14,69	0,875
	Exitus	2	16,57	5,95	
Troponin	Discharged	189	4,97	16,65	0,814
	Exitus	4	6,95	11,77	
CK	Discharged	188	3,09	6,36	0,824
	Exitus	4	2,38	1,10	
Glucose	Discharged	197	96,12	25,52	0,939
	Exitus	5	97,00	13,01	
Urea	Discharged	197	23,42	9,57	0,786
	Exitus	5	24,60	8,79	
Creatine	Discharged	197	0,39	0,17	0,855
	Exitus	5	0,38	0,16	

Mann-Whitney U test results showed a significant difference between the unit of admission and absolute lymphocyte count (ALC), CRP, LDH, alanine transaminase (ALT), aspartate transaminase (AST), ferritin, fibrinogen, D-dimer and length of hospitalisation (p < 0.001) (Table 2).

**Table 2:** The relationship between the hospitalizations of the patients and the laboratory results WBC; white blood cell, ANC; absolute neutrophil count, ALC; absolute lymphocyte count, CRP; C reactive protein, LDH; lactate dehydrogenase, ALT; alanine transaminase, AST; aspartate transaminase, CK; creatine kinase

	Place of Hospitalization	N	A	SD	p
WBC	COVID Pediatric Clinic	184	7,20	3,52	0,868
	COVID Intensive Care	16	7,73	5,07	
ANC	COVID Pediatric Clinic	184	3,50	2,66	0,411
	COVID Intensive Care	16	4,95	4,13	
ALC	COVID Pediatric Clinic	184	2,86	2,28	0,026
	COVID Intensive Care	16	1,82	1,33	
CRP	COVID Pediatric Clinic	182	1,02	3,33	0,001
	COVID Intensive Care	16	6,62	7,03	
LDH	COVID Pediatric Clinic	183	266,66	102,27	0,001
	COVID Intensive Care	14	544,36	257,72	
ALT	COVID Pediatric Clinic	195	33,17	19,85	0,001
	COVID Intensive Care	16	52,95	21,35	
AST	COVID Pediatric Clinic	196	21,86	54,72	0,003
	COVID Intensive Care	16	28,63	17,78	
Ferritin	COVID Pediatric Clinic	171	102,92	268,03	0,001
	COVID Intensive Care	14	923,17	1226,04	
Fibronogen	COVID Pediatric Clinic	181	244,32	106,85	0,001
	COVID Intensive Care	15	376,24	182,95	
D-Dimer	COVID Pediatric Clinic	177	1,77	6,45	0,001
	COVID Intensive Care	12	1,29	0,77	
Hemoglobin	COVID Pediatric Clinic	182	12,58	1,65	0,328
	COVID Intensive Care	16	12,03	1,91	
Platelet	COVID Pediatric Clinic	182	275,77	110,93	0,402
	COVID Intensive Care	16	273,06	133,95	
Vitamin D	COVID Pediatric Clinic	74	18,22	15,01	0,661
	COVID Intensive Care	6	17,64	6,66	
CK	COVID Pediatric Clinic	181	3,13	6,47	0,991
	COVID Intensive Care	11	2,24	1,34	
Glucose	COVID Pediatric Clinic	186	94,60	20,55	0,092
	COVID Intensive Care	16	114,06	54,54	
Urea	COVID Pediatric Clinic	186	23,38	9,45	0,883
	COVID Intensive Care	16	24,18	10,67	
Creatine	COVID Pediatric Clinic	186	0,40	0,17	0,279
	COVID Intensive Care	16	0,35	0,18	
Hospital stay	COVID Pediatric Clinic	203	3,47	2,67	0,001
	COVID Intensive Care	16	7,94	4,94	

## Discussion

Coronavirus is one of the major microbiological disease agents in animals and humans. At the end of December 2019, a novel type of coronavirus was defined in a pneumonia epidemic in China’s Wuhan city of the Hubei province, spreading rapidly since then and resulting in a worldwide pandemic [6]. In January 2020, the World Health Organization defined the agent in patients presenting with fever, cough, respiratory difficulty, nausea and diarrhoea as a novel coronavirus (2019-nCoV), which was not detected in humans before. The disease was later denominated as COVID-19. As a result of its close genetic resemblance to SARS-CoV, the virus was named SARS-CoV-2. Although the disease was initially limited to the Wuhan South China sea products market workers, it rapidly spread worldwide, compelling the World Health Organization to declare COVID-19 a pandemic as of 11 March 2020 [7].

Studies and data published on COVID-19 mainly focus on adults due to a greater prevalence and increased severity in that population segment because the paediatric population was reportedly rarely examined and tested in the early phases of the pandemic, limiting the data on the number of paediatric cases [8].

According to a study in Italy in 2020, 13.3% of patients treated for COVID-19 were hospitalised, and only 3.5% required intensive care [9]. In a 2019 study from Spain analysing the data of 365 paediatric COVID-19 patients, 66.7% were hospitalised for treatment, and only 9.1% of these patients were transferred to the intensive care unit [10]. In our study, a large majority of patients (92.7%) were admitted to the COVID-19 paediatric clinic, and a lesser percentage (7.3%) was admitted to the COVID-19 paediatric intensive care unit.

CRP is a highly sensitive protein with an increased plasma concentration during myocardial infarction, stress, trauma, infection, inflammation, surgery and neoplastic proliferation. The inflammation-based increase occurs within six to 12 hours and reaches its maximum by the 48th hour. It increases and decreases before other acute phase reactants. It does not indicate the cause of inflammation and is used during the course of inflammation. CRP increased particularly in 75-93% of severe COVID-19 patients [11]. We found that the CRP levels showed a significant difference in mortality (p < 0.001), with the deceased patients presenting significantly higher CRP levels.

Studies have shown that LDH levels increased in COVID-19 patients. LDH catalyses the last step of aerobic glycolysis (i.e., the reversible conversion of pyruvate to lactate) [12]. LDH reference ranges are initially high in children but gradually decrease during childhood. LDH increases in myocardial infarction, haemolytic anaemia, megaloblastic anaemia, hepatic disorders and progressive muscular dystrophy, and it is also used for the follow-up of non-Hodgkin lymphoma and leukaemia. In a study conducted on 140 COVID-19 patients from China in 2019, LDH appeared to be one of the most important predictors of intensive care unit admission [13]. LDH increases are prevalent in cases of COVID-19 treated in the intensive care unit, showing a poor prognosis. We observed statistically significant LDH levels were 16 times higher in our patients admitted to the COVID-19 intensive care unit compared to patients admitted to the ward. Moreover, analysing according to the LDH levels, we found significantly different mortality rates. Our study also demonstrated that the discharged patients had significantly lower LDH levels than those who died.

A study of 43 patient series reported that patients with severe COVID-19 disease had significantly higher fibrinogen levels. COVID-19 is associated with a rise in the inflammatory markers like D-dimer, fibrinogen and pro-inflammatory cytokines [14,15]. The fibrinogen levels were approximately two times higher in the deceased patients than in the discharged ones in our study.

## Conclusions

It is important to identify in terms of protection that may deteriorate in COVİD-19 patients. Early detection of these patients will reduce mortality. Patients with higher CRP, LDH, ferritin and fibrinogen levels had a higher risk of being admitted to the intensive care unit and dying, which makes it vital to foresee the course of COVID-19. A rapid and accurate treatment will save lives in these patient groups.
